# Surface Relief Modulated Grating in Azo Polymer—From the Tailoring of Diffraction Order to Reshaping of a Laser Beam

**DOI:** 10.3390/ma15228088

**Published:** 2022-11-15

**Authors:** Anna Kozanecka-Szmigiel, Aleksandra Hernik, Katarzyna Rutkowska, Jolanta Konieczkowska, Ewa Schab-Balcerzak, Dariusz Szmigiel

**Affiliations:** 1 Faculty of Physics, Warsaw University of Technology, 75 Koszykowa Str., 00-662 Warsaw, Poland; 2 Centre of Polymer and Carbon Materials, Polish Academy of Sciences, 34 M. Curie-Sklodowska Str., 41-819 Zabrze, Poland; 3 Łukasiewicz Research Network-Institute of Microelectronics and Photonics, al. Lotnikow 32/46, 02-668 Warsaw, Poland

**Keywords:** azo polymer, diffraction grating, surface relief grating, holographic recording

## Abstract

Among light-responsive materials for photonics, azo polymers occupy an important position due to their optical response and the related concepts of consecutive applications. However, scientific insight is still needed to understand the effects of irradiation on the modification of the azo polymer structure and the effect of this modification on incoming probing light. In this work, we report on a surface relief grating with a maximum depth of a record-high value of 1.7 µm, inscribed holographically in a custom synthesized glassy azo polymer belonging to the poly(ether imide) family. We show that the specifically deformed polymer, forming an amplitude-modulated relief grating, has a unique dual effect on an incoming light beam of different diameters. When illuminated by a narrow probe beam, the structure acts as a variable-depth grating, enabling a continuous tuning of the diffraction efficiencies in the entire theoretically predicted range and, thus, generating or eliminating diffracted waves of specified order. Alternatively, when illuminated by a wide probe beam, the whole structure acts as an optical component reshaping the Gaussian light intensity profile into the profiles resembling the squares of Bessel functions of the zeroth- or higher orders. Moreover, a physical justification of the effects observed is provided.

## 1. Introduction

Diffraction gratings are essential optical components widely used in spectroscopy, telecommunication, and laser systems for performing either a spectral analysis of light coming from an unknown source or beam splitting, combining, pulse compression, or stretching. The gratings may be fabricated using mechanical ruling or holographic technique, and their important parameter, i.e., the diffraction efficiency, may be controlled by the shape and depth of the grating grooves. In particular, in the case of a holographic grating having a sinusoidal groove profile, the diffraction efficiencies of the successive orders are described by squares of Bessel functions of the first kind of order corresponding to given diffraction order [1,2].

For many years azobenzene-containing polymers have been scientifically important materials showing the great wealth of the photoinduced phenomena that comprise the basis of the still-emerging concepts of novel applications [3,4,5,6,7,8]. The photoresponsive behavior of azo polymers arises from two microscopic processes appearing under irradiation with a linearly polarized light (typically of the wavelength from the UV–blue spectral region), that is, selective light absorption by a rod-like trans azo molecule and reversible trans-cis isomerization reaction [9,10]. Irradiation of an azo polymer layer with linearly polarized excitation light results, thus, in the generation of optical birefringence with the axis perpendicular to the polarization direction. In the case of irradiation with a light interference pattern (usually with the so-called polarization pattern, obtained with two orthogonally polarized coherent beams), both bulk birefringence grating and surface relief grating (SRG) is often generated, the second of which has a form of sinusoidal modulations of a sample thickness [11,12]. As the spacings of the sinusoidal modulations are of a few micrometers and modulation depths are of even one micron (in amorphous azo polymers), the relief structures may act as efficient gratings [13,14,15] redirecting a significant fraction of incident optical power away from the original propagation direction. That grating efficiency is experimentally determined already during sample irradiation with an interference pattern by measuring the intensity of another beam, i.e., the probe beam of the wavelength outside the azo polymer absorption band, diffracted from the grating into the first order. When irradiating the sample with three light beams (i.e., two writing and one probe beam), scientific attention has been naturally focused on the choice of exact excitation conditions leading to the generation of deep deformations and maximization of the first-order diffraction efficiency signal (η_+1_). In particular, the polarization state and the crossing angle of the two interfering beams are among these most crucial excitation conditions. Simultaneously, almost no attention has been paid either to the effect of the probe beam size on the experimentally observed SRG growth dynamics or on the cross-sectional intensity profiles of the generated diffracted beams. Only very recently, Jelken et al. [8,16] have presented their studies showing that the size of the probe beam relative to the size of the writing beams should be considered when interpreting the diffraction efficiency signals. The authors also reported on the fine structure of the first order diffraction spot profiles, i.e., the ring-shaped features (“the donut” or “the Saturn-like” structures) observed with an increase in the modulation depth when using a probe beam with a diameter similar to the SRG size.

It is widely recognized that apart from excitation conditions, the efficiency of SRG formation depends on the chemical structure of an azo polymer (in particular, the structure of the polymer main chain and the azobenzene chromophore, a type of a linkage between the chromophore and the main chain, or a polymer molecular weight) [3,17,18]. Unlike the flexible chain polymers (e.g., these from the methacrylate family), azo polymers possessing an imide group in the main chain are characterized by a high glass transition temperature, often above 200 °C. This property ensures thermal stability of the optically induced birefringence or SRGs, which is essential in practical applications; however, on the other hand, it does not correlate with an ability to form deep surface deformations [17,19]. Typically, the maximum achieved amplitudes of SRGs in azo polymers from the imide family are a few tens of nanometers leading to the diffraction efficiencies of the first order (η_+1_) of the order of 1% [19,20,21], though, under a pulsed UV excitation, surface deformations of 240 nm could also be formed [22].

Recently, we have observed the inscription of an unusually deep SRG (i.e., 260 nm) in a custom synthesized azo poly(ether imide) under a holographic 442 nm continuous-wave irradiation and measured η_+1_ of 20% [23]. Encouraged by these findings, we devote this work to investigating the formation of even deeper reliefs in this polymer and characterizing their topographic features in detail. Moreover, motivated by the study of Jelken et al. [16] and a willingness to fill the gap in the literature data, we focus our attention on the aspect of the size of a probe beam in examining the diffraction properties of the generated relief structures. We analyze the two sets of data gathered when using probe beams with very different spot diameters, either small or wide (comparing with the SRG area), and explain optical power distribution among diffracted beams as well as the origin of their specific spatial intensity profiles. We demonstrate that achieving extraordinary deep SRGs leads to unusually large light phase modulations, which in particular, results in suppression of the zeroth or higher diffraction orders and redirection of the light intensity in the other allowed directions.

## 2. Materials and Methods

**Figure 1:**
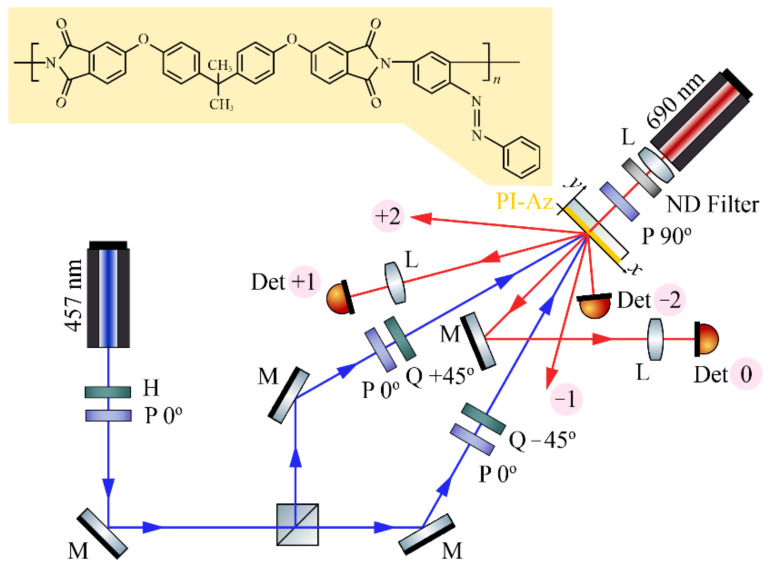
Experimental setup for recording SRG in PI-Az and monitoring its formation dynamics; P—polarizer, M—mirror, H—half-wave plate, Q—quarter-wave plate, L—lens, ND Filter—neutral density filter, and Det 0, Det +1, and Det −2—detectors measuring the optical power of the 0th, +1st or −2nd order diffracted beam, respectively. The chemical structure of the studied PI-Az polymer is presented in the inset.

The chemical structure, synthesis route and physicochemical properties of the investigated side-chain azobenzene poly(ether imide) PI-Az were described recently [24]. The polymer contained one unsubstituted azobenzene moiety per a repeating unit, attached to the backbone between the imide groups in such a way that one of the azobenzene aromatic rings was a part of the main chain (see the inset in Figure 1). PI-Az possessed an amorphous structure and a relatively high glass transition temperature of 173 °C [24].

The samples used in the SRG recording experiment were prepared in the form of the PI-Az layer on a glass substrate. The layers were obtained by dissolution of the azo polymer in N-methyl-2-pyrrolidone (0.1 g of polymer per 5 mL of the solvent), casting onto the substrates, and then drying in a vacuum at 100 °C overnight. The thickness of the obtained layers was 2.4–3.4 μm, measured with a Dektak XT stylus profiler.

Figure 1 presents the scheme of an experimental setup used for the holographic recording of the SRG in PI-Az and monitoring its formation process over time.

An ultra-low noise 457 nm DPSS laser was used for SRG inscription. The intensity of each of the two interfering beams was 105 mW/cm^2^, while the size (1/e^2^) of their elliptical spots at the sample plane was 2.0 × 1.5 mm. The beams crossed on the polymer layer at an angle of 3°, forming an interference pattern with a ca. 8.7 µm period (according to the formula: Λ = λ/(2sin(θ/2)), where λ is the writing wavelength and θ is the intersection angle) [15]. The sets of a polarizer and quarter-wave plate components, with the waveplates’ axes oriented at +45° or −45° to the vertical direction, were used to obtain right- or left-handed circular polarization of the interfering beams. In the intentionally chosen small recording angle geometry, the optical field in the beam overlapping area was characterized by a pure linear polarization state with an azimuth continuously rotating over one modulation period (along the *x*-direction) and by the lack of modulations in the total intensity distribution [25]. For monitoring the grating buildup process over time, a horizontally polarized 690 nm probe light from a diode laser with adjustable spot size was used. The laser was mounted behind the sample (i.e., from the opposite side of the incidence plane of the 457 nm beams) due to a very compact recording geometry. The red beam was focused to the size as small as 110 μm at the sample plane illuminating 12 grating grooves. The beam optical power was suppressed to 19 µW with the aid of neutral density filter. Three silicon photodetectors were used to measure the optical power of the 0th, −1st, and +2nd diffraction orders in 1 s time intervals during 3.5-h holographic exposure of the sample. In order to focus the diverging 0th and −1st order diffracted beams on the detectors’ surfaces, additional lenses were inserted in the optical paths of the beams (Figure 1).

For another PI-Az sample area, which was exposed to polarization pattern for up to 6 h, a detailed characterization of diffraction properties of the inscribed SRG was performed in two different measurements arranged with either a narrow or wide 690 nm probe beam. In the first case, the PI-Az sample was mounted on the *x*-axis translation stage so that the beam focused to a 110 μm diameter was incident on one of the edges of the inscribed structure. Then, while translating the sample with respect to the 690 nm spot every 20 μm, the optical power of the 0th and +1st diffraction orders was recorded by photodetectors till the probe beam illuminated the opposite edge of the SRG. The optical power of other diffraction orders, i.e., +2nd, +3rd, and +4th was measured in translation steps of 50 or 100 μm starting from the edge of the SRG and ending at its center. Diffraction efficiencies (η) were calculated as the ratios of the optical power directed into a particular order to the incident power. In the second measurement, arranged with a wide probe beam, the SRG was illuminated with a ca. 2.5 mm-diameter beam collimated with a two-lens system. A CMOS laser beam profiling camera (Beamage, Gentec-EO) recorded the diffracted beam spots behind the sample. The horizontally polarized probe beams at normal incidence were applied in each type of measurement.

A Dektak XT stylus profiler was used to examine the topography profile of the irradiated azopolymer surfaces along the *x*-direction. The diamond-tipped stylus had a 0.2 µm radius, a stylus force of 1 mg, and a traversing resolution of 0.07 µm.

## 3. Results

**Figure 2:**
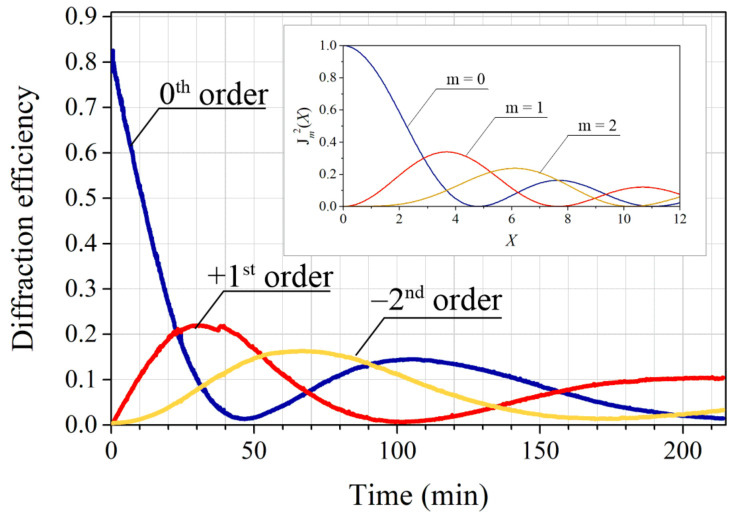
Evolution of the 0th, +1st, −2nd order diffraction efficiencies of the surface grating continuously formed in the PI-Az layer during irradiation with a light polarization pattern. The squares of the mathematical Bessel functions of the 0th, 1st, and 2nd order shown in the figure inset.

Irradiation of the PI-Az sample with a light polarization pattern in the setup presented in Figure 1 led to a formation of a diffraction grating with period equal to the arranged polarization pattern spacing, which was confirmed by the measurement of the first-order diffracted angle (±4.5° for 690 nm light). Just after turning on the writing beams, the intensity of the non-diffracted 690 nm light started to decrease, accompanied by an increase in the first-order diffraction intensity; the generation of the second-order diffracted beam was detected correspondingly later. Figure 2 presents the temporal evolutions of the determined diffraction efficiencies of the 0th, +1st, and −2nd orders during 3.5 h of the holographic recording.

A striking feature of the plots is the shape of all detected curves resembling the squared Bessel functions of the zeroth, first, and second order, respectively. The curves were characterized by well-resolved peaks and the minima reaching practically zero, indicating huge values of phase modulation amplitude introduced by the continuously inscribed structure. To our knowledge, the plots of time-evolution of diffraction efficiency signals having fully developed maxima and minima at zero values have not been reported in the literature for glassy azo polymers.

**Figure 3:**
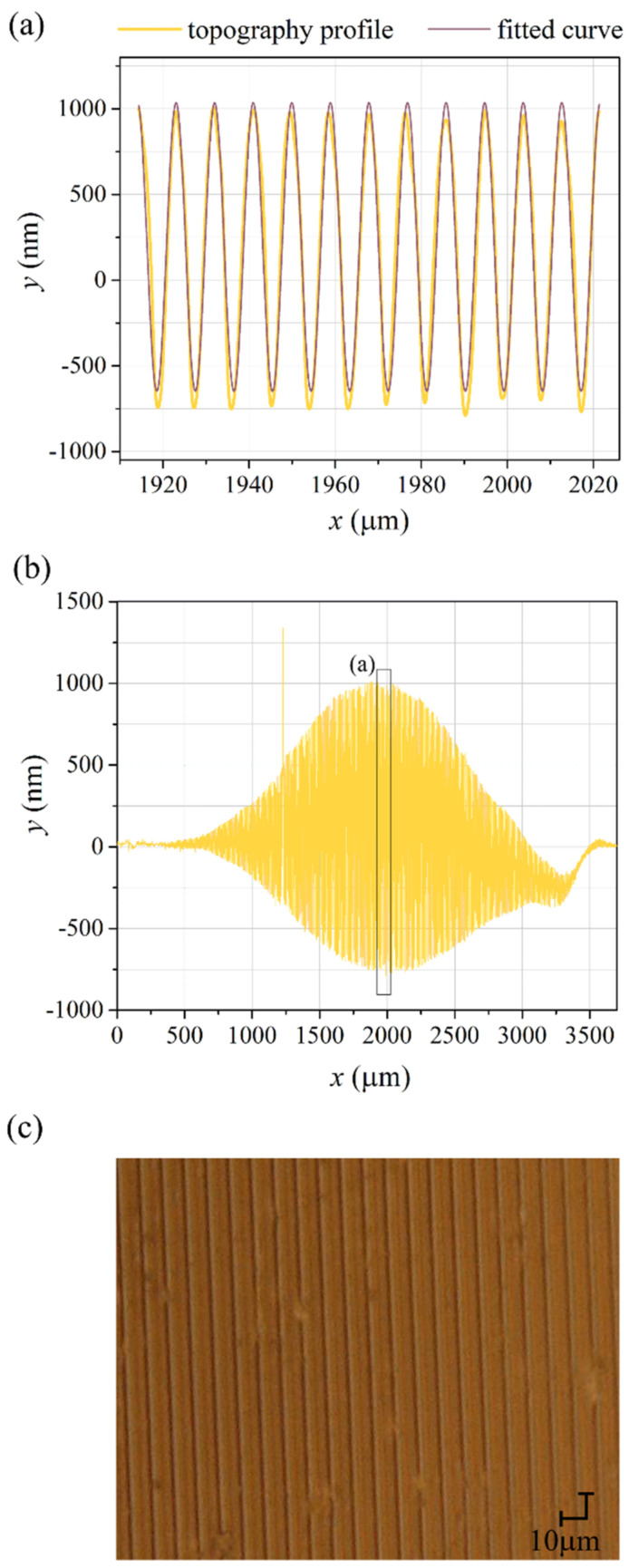
The topography profile (in dark yellow) of the PI-Az surface measured in the *x*-direction along (**a**) a ca. 100 µm distance in the center of the light-exposed region, together with a fitted sine function (in violet), (**b**) the whole light-exposed area. (**c**) The optical microscopy image in reflected light, referring to the center of the generated SRG. Layer thickness at the irradiated area was 3.3 µm.

The Bessel function shapes of the detected curves are expected for a sinusoidal phase grating; nevertheless, a closer look at the figure revealed deviations of the curve slopes compared to the corresponding mathematical functions (see inset in Figure 2). Moreover, the first maximum in the +1st order diffraction efficiency curve had a lower value compared to that of the $J_{1}^{2}$ function, while simultaneously, the value of the following maximum agreed well with the corresponding theoretical one. The former discrepancy (i.e., the curve shapes) could be explained by a nonlinear grating inscription rate: the fastest at the initial irradiation periods and then getting slower. The latter discrepancy, i.e., the undervalued maximum of the first peak might be associated with diffraction on the grating, having the area smaller than the size of the probe beam [26]. Then the correct value of the second maximum in the +1st order curve would be justified by diffraction on the grating with a bigger size, at least that of the probe beam [26]. It is worth pointing out that the maximum first order diffraction efficiency arising from the bulk birefringence grating was estimated to be below 0.5% [23], i.e., significantly lower than the maximum values observed for that diffracted order. The examination of the PI-Az sample topography after the holographic recording revealed an unusually deep relief of 1500 nm height inscribed at the center of the irradiated area. Another holographic recording performed for a much longer time (6 h) led to the formation of the relief structure having 1720 nm of the maximum depth. In Figure 3a, the yellow curve shows the topography profile taken in the central area of the 6-h exposed surface (along 12 grating grooves), while the violet curve represents the fitted sine function. The excellent quality fitting confirmed the sinusoidal grating inscription according to the expectations.

Figure 3b presents the PI-AZ surface profile taken over a few millimeter-length along the *x*-axis. The most striking finding from the obtained topography scan is that the inscribed SRG took a form of an amplitude-modulated structure with upper and lower bell-shaped envelopes reflecting Gaussian light intensity distribution at the irradiation area. Moreover, the deformations of a sinusoidal profile were generated in the region spanning the length of 3.1 mm, which is significantly longer than the 1/e^2^ size of the *x*-axis of the elliptical interference spot. The lowest amplitude of these grooves was 30 nm; even more minor deformations were found at the far edges of the relief; however, their profile was no longer sinusoidal. A shallow but visible relief at the edges proved that the surface structuring does not require any threshold light intensity, but rather a light fluence (intensity multiplied by time) is essential. Figure 3c presents the optical microscopy image in reflected light, referring to the center of the generated SRG. The regular pattern characteristic for a one-dimensional grating and few defects in the PI-AZ layer were visible. The pattern spacing of 9 μm agreed with the periodicity of the surface deformation found in the topography measurements with a stylus profiler.

Though applied excitation conditions may affect the efficiency of the SRG inscription process, the maximum SRG depth of 1.7 μm observed in PI-Az is an absolute record for azo polyimides and, to our knowledge, for glassy azo polymers in general (Table 1).

The strong dependence of the SRG formation process on the irradiation conditions is visible when comparing the maximum depth of the SRG inscribed in PI-Az in this and the previous study (Δ*d_max_* = 260 nm) [23]. Generation of a much deeper SRG arises from significantly longer irradiation time (6 h vs. 1.5 h), higher beam intensity of the interfering beams (105 mW/cm^2^ vs. 50 mW/cm^2^ per each beam), and an excitation laser source (low noise DPSS 457 nm laser vs. 442 nm gas He-Cd laser).

**Figure 4:**
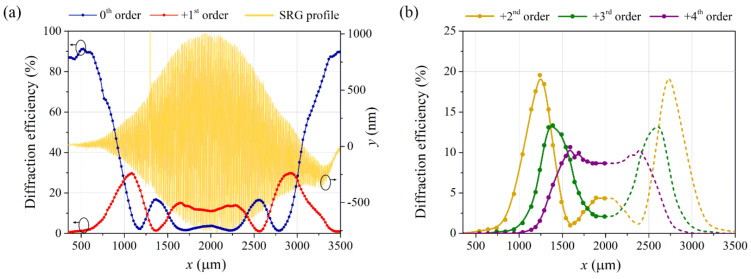
The diffraction efficiencies of the subsequent orders measured while scanning the SRG with a narrow 690 nm probe beam (**a**) 0th and +1st order, (**b**) +2nd, +3rd and +4th order. The dotted lines in (**b**) are drawn as mirror reflected plots to the data gathered up to *x* = 2000 µm.

The diffraction properties of the whole generated SRG were probed using a 690 nm beam either focused to 110 µm spot size or collimated to a diameter comparable to the size of the relief structure. Figure 4a illustrates the 0th and +1st order diffraction efficiency values determined while scanning a focused 690 nm beam across the SRG (from one grating edge towards the center to the second edge). The detected η values have been merged for visualization purposes with the SRG topography profile (Figure 3b).

As it can be noticed, when the probe beam was incident on either of the relief edges, i.e., at positions with a low modulation depth of ca. 30 nm, it was transmitted through the sample practically undeflected. The zeroth order diffraction efficiency was 90%, then, and only very low-intensity first order diffracted light waves were present behind the sample. With a change of the beam position to the relief center, the values of both zeroth and first diffraction efficiencies underwent non-monotonous variations. What should be stressed here is that the variations resembled the plots of squares of the zeroth and first order Bessel functions. In the central region of SRG (i.e., around *x* = 2000 µm), the values of diffraction efficiencies varied only slightly as a function of *x* due to little changes in the relief height. Scanning the sample with the probe beam from the SRG central area to the opposite edge resulted in the diffraction efficiency plots symmetrical to the vertical axis at *x* = 2000 µm. It is worth noting that the minima of both zeroth and first diffraction efficiency signals reached practically zeros and the successive maxima were close to 17% for the zeroth order signal and 30% or 14% for the first order signal, respectively. The measured diffraction efficiencies were thus such as the theoretically predicted for the sinusoidal phase grating. Figure 4b shows analogous plots as in Figure 4a but obtained for the second, third and fourth diffracted orders. Again, the obtained function plots resemble the squares of the corresponding Bessel functions.

**Figure 5:**
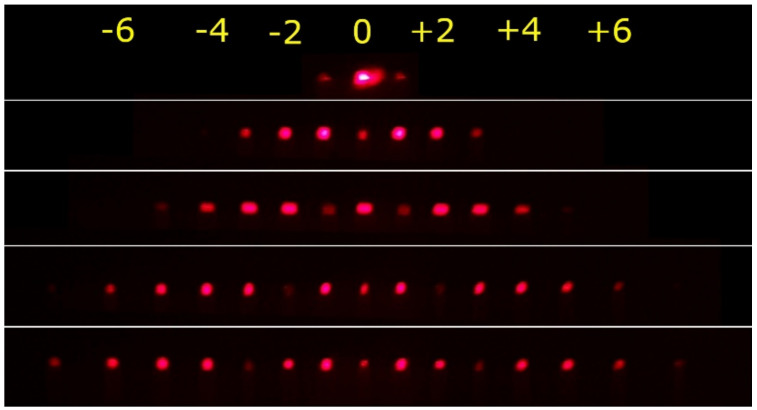
Photographs of the diffraction patterns observed on the screen behind the PI-Az sample. The photos were taken when translating the sample along the x direction so that the red probe beam was normally incident on the selected positions on the inscribed SRG. Apart from the top photograph, the following ones correspond to the points of incidence where the intensity of the 0th, 1st, 2nd and 3rd diffraction orders vanished, respectively. The top and bottom rows correspond to the beam position at the SRG edge and center, respectively.

In Figure 5, photographs of the diffraction patterns taken behind the PI-Az sample while scanning the focused probe beam along the SRG as described above (i.e., from the SRG edge to the center) are given. The top pattern was observed on the screen when illuminating the edge of the SRG. The patterns in the subsequent rows were observed when illuminating the specific positions (along the *x*-axis), where the zeroth, first, second and third order diffraction efficiencies were minimized. The inscribed structure generated multiple diffraction orders allowing complete and selective suppression of the diffraction orders from zeroth up to the third.

**Figure 6:**
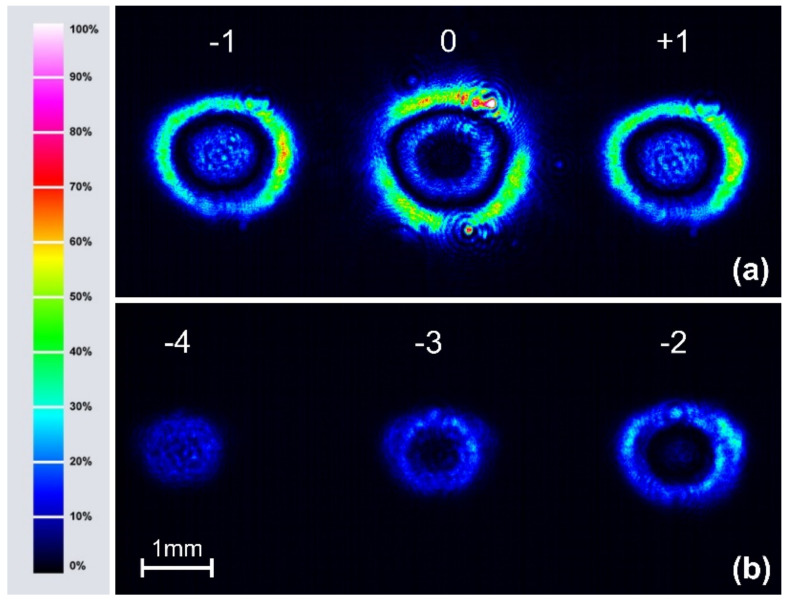
The cross-sectional intensity distribution of 690 nm diffracted beams of (**a**) the 0th, +1st and −1st order, (**b**) the −2nd, −3rd and −4th order observed behind the PI-Az sample with the aid of a CMOS camera. The colour legend shows the intensity levels of the diffracted spots. The scale bar refers to both pictures.

Figure 6 illustrates the diffraction pattern generated by the inscribed SRG when using the collimated 690 nm probe beam of a diameter matching the size of the structure. In this case, the unique non-Gaussian cross-sectional intensity profiles of the diffracted beams were found. The 0th, ±1st order diffraction spots detected by a CMOS camera placed behind the PI-Az sample are shown in Figure 6a, while the −2nd, −3rd and −4th order diffraction spots are presented in Figure 6b. The concentric “ring” structure of the diffraction spots was seen for up to the 3rd order. In particular, the zeroth order spot was composed of a dark central area and the bright outer “ring” with a brightness higher than the inner one. Simultaneously, a bright central area, a dark ring and another high-brightness outer ring characterized both the +1st and −1st order diffraction spots. It should be noted here that the cross-sectional intensity distributions of all the positive diffraction orders were the same as the negative ones.

A fine structure in the spatial profile of the first order diffraction spot was recently reported by Jelken et al. [16]. While monitoring the SRG holographic buildup process with a wide probe beam, the time-evolution of the first order diffraction spatial profile from a Gaussian through a “donut” one (with a dark center) to a ring structure with a bright center was found. The authors pointed out that the SRG did not have a constant modulation amplitude over the inscribed area and showed a schematic representation of the inscribed structure. The Raman–Nath-based model was also proposed to explain the effect observed.

**Figure 7:**
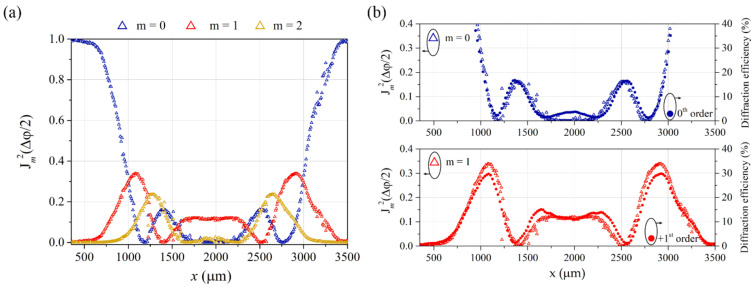
(**a**) The squares of the Bessel functions, $J_{m}^{2}$((∆φ(*x*))/2), for *m* = 0, *m* = 1, and *m* = 2 calculated based on the local SRG amplitude at *x* along the irradiated area. (**b**) The squares of the first two Bessel functions *m* = 0, *m* = 1 (taken from Figure 7a) merged for comparison with the 0th and +1st order diffraction efficiencies measured while translating a narrow probe beam with respect to the SRG (taken from Figure 4a).

In this study, justification of the effect of ring structure in the diffraction spots has been provided by experimental data on diffraction efficiencies probed by the narrow beam (as presented in Figure 4a,b). Simultaneously, knowing the whole-length surface topography profile after irradiation allowed for a theoretical justification of the effect. Using the formulae: $\Delta \varphi \left(x\right)=\frac{2\pi }{\lambda }d\left(x\right)\cdot \left(n-1\right)$, where *d*(*x*) is a local peak-to-valley SRG depth, and *n* is the effective refractive index of PI-Az, the local phase modulation amplitude of the λ = 690 nm light passing through the inscribed structure was determined [2]. Figure 7a presents the calculated squares of the Bessel functions of the 0th, 1st and 2nd order: $J_{m}^{2}\left(\frac{\Delta \varphi \left(x\right)}{2}\right)$, *m* = 0, 1, 2 at different x positions along the whole SRG, taking *n* equal to 1.7. In Figure 7b, the two calculated Bessel functions $J_{0}^{2}$ and $J_{1}^{2}$ are presented together with the measured 0th and 1st order diffraction efficiencies (from Figure 4a) for comparison.

The excellent agreement between the two data sets regarding extrema positions and values is evidenced. It is worth noting that the determined values of $J_{m}^{2}\left(\frac{\Delta \varphi \left(x\right)}{2}\right)$ are diffraction efficiencies achieved when considering the inscribed SRG alone, while diffraction efficiencies obtained in the optical measurement result from the presence of both the relief and bulk birefringence gratings. The agreement between the corresponding plots proves that light diffraction on the PI-Az grating was dominated by the contribution from the surface relief structure and not the bulk birefringence grating. Moreover, comparing the two data sets (as done in Figure 7b) allowed for estimating the PI-Az effective refractive index at the probe wavelength. It has been verified that the effective refractive index values differing by 0.1 from assumed *n* = 1.7 led to a much poorer agreement between the sets of plots.

The ring structure of the diffraction spots (shown in Figure 6) appearing when using a wide probe beam arises from locally different phase retardations accumulated by a passing light. The central parts of each spot originate from the relief center, while their outer regions originate from the relief edges. In particular, the darkened central area of the 0th order spot is due to a huge phase modulation at which a second minimum of the Bessel function $J_{0}^{2}$ appears. The outer high brightness ring of the zeroth order spot is due to low phase modulation and corresponding high transmittance of the incident light. Low phase modulation at relief edges and thus low values of the squared higher orders Bessel functions also explain a smaller diameter of the higher-order diffraction spots than the diameter of the zeroth order outer ring.

## 4. Conclusions

We have demonstrated that the custom synthesized azo poly(ether imide) PI-Az exhibits an extraordinary ability to form SRGs. The maximum deformation depth inscribed in the PI-Az layer during holographic recording with two 457 nm laser beams of opposite circular polarization was 1.7 μm, an absolute record for azo polyimides and glassy azo polymers in general. Very deep sinusoidal surface modulations resulted in large phase retardations of passing light and allowed for diffraction efficiencies of specific orders non-achievable before for glassy azo polymers.

When monitoring the holographic inscription process with the beam as narrow as a few grating grooves, it was possible to detect the diffraction efficiency curves evolving with time in accordance with the Bessel function plots. It was proposed that the deviations between the characteristics of experimental and theoretical curves might be used as an indicator of the changes in SRG built-up dynamics and in the SRG size at a given instance.

Holographic recording performed with interfering beams of a Gaussian intensity profile led to the formation of the SRG having variable groove depth. The PI-Az surface topography scan taken along the whole irradiated area revealed an amplitude-modulated structure with double-sided envelopes of the SRG peaks and valleys appearing above and below the initial PI-Az layer level, respectively. The inscribed structure offered a unique dual use depending on the size of the probe beam: either being a grating with continuously regulated diffraction efficiency or an optical component reshaping the incoming Gaussian intensity profile. In the first case, by translating a narrow beam with respect to the sample, it was possible to fully maximize or minimize the intensity of the diffracted beams of the desired order (from the zeroth up to the third one) and thus, switch them on and off. In the second case, using a wide beam it was possible to generate the diffracted light waves characterized by a ring structure in the cross-sectional intensity distribution.

Our findings show the great applicational potential of the studied azo poly(ether imide) in the area of photonics, as well as document the importance of the exact size of the probe laser beam in studying the diffraction properties of deep SRG formed in azo polymers during and after recording.

## Figures and Tables

**Table 1:** Maximum depth (Δ*d_max_*) and the maximum first-order diffraction efficiency (η_+1_) of SRG reported for different glassy azo polymers.

Glassy Azo Polymer	Δ*d_max_*/η_+1_
Azo polymer PDR13A	900 nm/42% [13]
An epoxy-based glassy azobenzene polymer	250 nm/22% [15]
Poly [1-[4-(3-carboxy-4-hydroxyphenylazo)benzenesulfonamido]-1,2-ethanediyl, sodium salt] (Pazo) Azocarbazole-based polyimide	ca. 500 nm/1.5% * [16] 50 nm/0.25% [19]
Azobenzene functionalized poly(amide-imide)	80 nm/1.55% [20]
Azo-dye bearing poly(esterimide)	68 nm/0.7% [21]

* η_+1_ measured for the beam reflected from glass substrate side only.
